# Adolescent depression and subsequent earnings across early to middle adulthood: a 25-year longitudinal cohort study

**DOI:** 10.1017/S2045796020000360

**Published:** 2020-04-29

**Authors:** Anna Philipson, Iman Alaie, Richard Ssegonja, Henrik Imberg, William Copeland, Margareta Möller, Lars Hagberg, Ulf Jonsson

**Affiliations:** 1 University Health Care Research Center, Faculty of Medicine and Health, Örebro University, Örebro, Sweden; 2 Department of Neuroscience, Child and Adolescent Psychiatry, Uppsala University, Uppsala, Sweden; 3 Department of Public Health and Caring Sciences, Uppsala University, Uppsala, Sweden; 4 Statistiska Konsultgruppen, Gothenburg, Sweden; 5 Department of Mathematical Sciences, Chalmers University of Technology and University of Gothenburg, Gothenburg, Sweden; 6 Vermont Center for Children, Youth and Families in the Department of Psychiatry, University of Vermont Medical Center, Burlington, VT, USA; 7 Karolinska Institutet Center of Neurodevelopmental Disorders (KIND), Centre for Psychiatry Research, Department of Women's and Children's Health, Karolinska Institutet & Stockholm Health Care Services, Stockholm County Council, Stockholm, Sweden

**Keywords:** Adolescents, depression, economic issues, epidemiology, mental health

## Abstract

**Aims:**

The few available studies on early-onset depression and future earnings offer ambiguous findings, and potential sources of heterogeneity are poorly understood. We examined the differences in adult earnings of males and females with and without a history of depressive disorder in adolescence, with specific focuses on (1) future earnings in clinical subtypes of adolescent depression; (2) the growth and distribution of earnings over time within these subgroups and (3) the mediating role of subsequent depressive episodes occurring in early adulthood.

**Methods:**

Data were drawn from the Uppsala Longitudinal Adolescent Depression Study, a community-based cohort study initiated in Uppsala, Sweden, in the early 1990s. Comprehensive diagnostic assessments were conducted at age 16–17 and in follow-up interviews 15 years later, while consecutive data on earnings for the years 1996 to 2016 (ages 20–40) were drawn from population-based registries. The current study included participants with a history of persistent depressive disorder (PDD) (*n* = 175), episodic major depressive disorder (MDD) (*n* = 82), subthreshold depression (*n* = 64) or no depression (*n* = 218) in adolescence. The association of adolescent depression with earnings in adulthood was analysed using generalised estimating equations. Estimates were adjusted for major child and adolescent psychiatric comorbidities and parental socioeconomic status. The indirect (mediated) effect of depression in early adulthood (ages 19–30) on earnings in mid-adulthood (31–40) was estimated in mediation analysis. The study followed the ‘STrengthening the Reporting of OBservational studies in Epidemiology’ (STROBE) guidelines.

**Results:**

Earnings across early to middle adulthood were lower for participants with a history of a PDD in adolescence than for their non-depressed peers, with an adjusted ratio of mean earnings of 0.85 (0.77–0.95) for females and 0.76 (0.60–0.95) for males. The differences were consistent over time, and more pronounced in the lower percentiles of the earnings distributions. The association was partially mediated by recurrent depression in early adulthood (48% in total; 61% for females, 29% for males). No reduction in earnings was observed among participants with episodic MDD in adolescence, while results for subthreshold depression were inconclusive.

**Conclusions:**

Our findings suggest that future earnings of adolescents with depressive disorders are contingent on the duration and natural long-term course of early-onset depression, emphasising the need for timely and effective interventions to avoid loss of human capital.

## Introduction

Early-onset depression is common worldwide, with an estimated life-time prevalence of 15% in late adolescence and a strong female preponderance (Merikangas *et al*., 2010). Depression in adolescence is linked to recurring episodes and other mental health conditions in adulthood (Johnson *et al*., 2018), with a particularly poor prognosis reported for persistent depressive disorder (PDD) (Jonsson *et al*., 2011). Adolescent depression is also associated with subsequent role impairment across several life domains (Clayborne *et al*., 2019), and the few available cost-of-illness studies suggest substantial direct and indirect societal costs (Bodden *et al*., 2018; König *et al*., 2019; Ssegonja *et al*., 2019).

A recent study showed that behaviours observed in children as young as 5 to 6 years can predict earnings in adulthood (Vergunst *et al*., 2019), underscoring that future financial circumstances can be rooted early in life. From the human capital approach, earnings reflect the individual's productivity and contribution to society (Berger *et al*., 2001). Loss of productivity leads to loss of income for the worker, and production-loss for the firm and society (Marcotte and Wilcox-Gok, 2001). To inform evidence-based policymaking and treatment planning, it is therefore important to have a clear picture of the long-term economic outcome of early-life depression. The relatively small number of longitudinal studies on adolescent depression and future earnings show ambiguous findings (Fletcher, 2013; Johar and Truong, 2014; Evensen *et al*., 2017; Clayborne *et al*., 2019). Prospective studies of adolescents assessed for depressive disorders have found small but non-significant adjusted associations (Weissman *et al*., 1999; Naicker *et al*., 2013; McLeod *et al*., 2016), or no association at all, between adolescent depression and future earnings (Lewinsohn *et al*., 2003). Studies focusing on self-reported depressive symptoms in adolescence, on the other hand, showed a reduction by 4–12% on earnings in young adulthood (Fletcher, 2013; Johar and Truong, 2014). In addition, it has been reported that self-reported internalising problems in adolescence are associated with reduced earnings in adulthood (age 30–36) with about 3%, with larger effects in the lower part of the earnings distribution (Evensen *et al*., 2017).

There are some important limitations to the existing body of research. First, with few exceptions (McLeod *et al*., 2016; Evensen *et al*., 2017), the outcome has been measured at an age when a substantial proportion of the population has not yet entered the labour market (e.g. <30 years) (Weissman *et al*., 1999; Lewinsohn *et al*., 2003; Fletcher, 2013; Naicker *et al*., 2013; Johar and Truong, 2014). Second, previous research has relied on self-reported information on earnings measured at a few time-points, which might not reflect the true earnings over time (Weissman *et al*., 1999; Lewinsohn *et al*., 2003; Fletcher, 2013; Naicker *et al*., 2013; Johar and Truong, 2014; McLeod *et al*., 2016). Third, it is not yet established to what extent the outcome is contingent on clinical characteristics of the disorder (e.g. duration and recurrence).

Here, we combined data from a relatively large Swedish cohort study, the Uppsala Longitudinal Adolescent Depression Study (ULADS) (Alaie *et al*., 2019), with register-based data on year-by-year earnings from late adolescence up to approximately 40 years of age. ULADS is based on in-person assessments using diagnostic criteria to ascertain specific subtypes of depressive disorders, including PDD, major depressive disorder (MDD) and subthreshold depression. Our aim was to investigate the differences in adult earnings of males and females with and without a history of depressive disorder in adolescence, with specific focuses on (1) future earnings in clinical subtypes of adolescent depression; (2) the growth and distribution of earnings over time within these subgroups and (3) the mediating role of subsequent depressive episodes occurring in early adulthood.

## Method

This study was based on ULADS, a longitudinal study initiated in Uppsala, Sweden, in 1991 (Alaie *et al*., 2019). The original study was approved by the Ethical Committee of Uppsala University. For each new phase of the study, ethical approvals have been obtained from the Regional Ethical Review Board in Uppsala, including the current phase (2015/449/1-2). Participants consented to future contact by providing their unique personal identity number (Alaie *et al*., 2019). The study follows the standard methodology of cohort studies and is reported according to ‘STrengthening the Reporting of OBservational studies in Epidemiology’ (STROBE) guidelines (von Elm *et al*., 2014).

### Study population and data collection

**Fig. 1.**
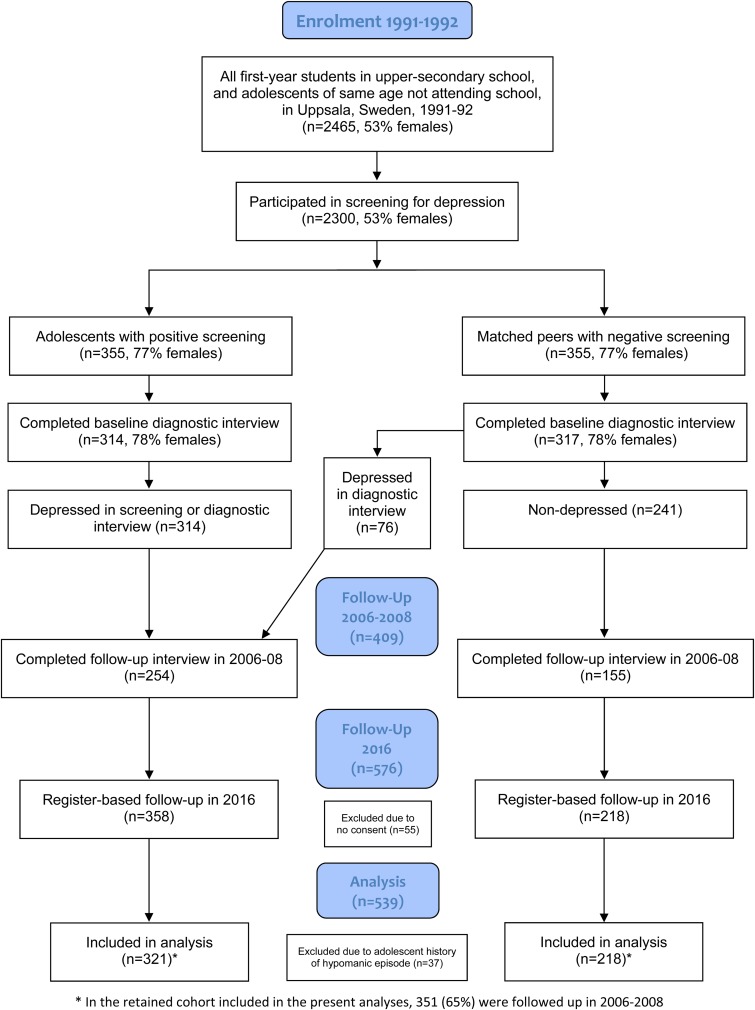
Flow diagram.

The source population comprised of all first-year students in upper-secondary school (aged 16–17) and adolescents of the same age-group that did not enrol in upper-secondary school during the academic year (*N* = 2465) (Olsson and von Knorring, 1999). A total of 2300 (93%) were screened for depression. The Beck Depression Inventory-Child (BDI-C) (Beck *et al*., 1961) and the Center for Epidemiological Studies-Depression Scale for Children (CES-DC) (Schoenbach *et al*., 1982) were used for the screening procedure. Positive screening was defined as BDI ⩾ 16, or CES-DC ⩾ 30 + BDI ⩾ 11, or a self-reported suicide attempt. Students with positive screening (*n* = 355) were invited to take part in a comprehensive face-to-face assessment including a blinded structured diagnostic interview, using the Diagnostic Interview for Children and Adolescents in the Revised form according to DSM-III-R for Adolescents (DICA-R-A) (Reich *et al*., 1982). For every student with positive screening, a same-sex classmate with negative screening, who was close in age, was invited to an identical assessment. Out of the 710 invited adolescents, 631 participated (Fig. 1).

In the years 2006–2008 (age 30–33), participants who previously had consented to continued contact (*n* = 609) were invited to a follow-up interview. A total of 409 individuals participated (65% of the original cohort).

Registry-data were obtained for 576 participants (91% of the original sample) as regards the period from 1996 to 2016 (i.e. up to age 40/41), excluding 55 former participants who either did not consent to future contact (*n* = 22) or who previously had refused extraction of individualised registry data (*n* = 33). While participants with comorbid conditions were included, participants with an early-onset manic/hypomanic episode (*n* = 37) according to the DICA-R-A assessments were excluded from the current study. The rationale for this was that bipolar disorder is classified separately from depressive disorders in the Diagnostic and Statistical Manual of Mental Disorders (DSM) (American Psychiatric Association, 2013), and could potentially affect future earnings in a way that differs from depressive disorders. Exclusion of this group left a total of 539 eligible participants. Details of the study population and procedure have been described elsewhere (Alaie *et al*., 2019).

### Measures

#### Adolescent depression

The subcategorisation of adolescent depression for the current study was created using information from the diagnostic assessment at baseline. The DICA-R-A is based on the DSM-III-R (American Psychiatric Association, 1987) criteria. In order to conform the prevailing classifications, the DSM-III-R diagnoses were converted to depressive disorders as defined by DSM-5 (American Psychiatric Association, 2013). The following definitions were used:

1. PDD: depressed mood occurring for most of the day, for more days than not, for at least 1 year. The disorder subsumes previous DSM-III-R and DSM-IV definitions of chronic MDD and dysthymic disorder (*n* = 175).
2. Episodic MDD: a current or life-time major depressive episode lasting shorter than one year (*n* = 82).
3. Subthreshold depression: a positive screening but no other past or current depressive disorder (*n* = 64).
4. No depression: negative screening, no past or current depressive disorder (*n* = 218).

#### Depression in early adulthood

Depressive episodes in early adulthood (ages 19–30) were measured retrospectively at follow-up using the Mini International Neuropsychiatric Interview 5.0.0 (MINI-PLUS) (Sheehan *et al*., 1998) in combination with a life-chart. In order to create a dichotomised mediator variable of clinical relevance, depression in early adulthood was either defined as a long episode (⩾6 months), or recurrent episodes (⩾2) from age 19 to 30.

#### Earnings

Information on earnings was harvested from the Longitudinal Integration Database for Health Insurance and Labor Market Studies (LISA) for all consecutive years following graduation from upper-secondary school (i.e. 1996–2016). LISA includes information on all individuals (⩾age 16) registered as residents of Sweden at the end of each calendar year, and is based on several national registries and administration systems. The database is updated annually with good coverage reported (Ludvigsson *et al*., 2019). Individual earning are a summed variable of gross income from employment and net income from active business.

The annual earnings were converted to the value of January 2019 using consumer price index (Statistics Sweden, 2019a). All earnings were converted from Swedish krona (SEK) to US Dollar (USD) using an approximated exchange rate of 1 SEK = 0.108 USD, as valid in January 2019.

#### Parental socioeconomic status

Parental education and income in 1990, retrieved from Statistics Sweden (Ludvigsson *et al*., 2019), were included as a proxy measure of socioeconomic status (SES) in adolescence. The highest achieved level of education of either parent, according to the Swedish Educational Terminology (SUN) (Statistics Sweden, 2019b), was divided into two categories: low (elementary school or upper-secondary school), and high (college or university). Both parents' disposable incomes were summed.

#### Comorbidity

Two major categories of childhood/adolescent psychiatric comorbidities were defined based on the DICA-R-A (Reich *et al*., 1982) assessments: disruptive behaviour disorders defined as a child or adolescent DSM-III-R diagnosis of conduct disorder, oppositional-defiant disorder and/or attention deficit/hyperactivity disorder; anxiety disorder defined as a child or adolescent DSM-III-R diagnosis of separation anxiety disorders, overanxious disorder and/or avoidant disorder.

### Missing data

There were two major sources of missing data regarding earnings: resettling abroad (*n* = 66) and mortality (*n* = 3). The mean total time residing abroad among those who had resettled was 7.3 years (SD = 6.0). For participants living abroad less than 6 months a specific year, an annual earning was estimated based on their earnings during the part of the year that they lived in Sweden. Participants who had been abroad more than 6 months of a particular year were categorised as missing for the specific year. Participants with missing values on parental education (*n* = 17) were assigned a low parental education level.

The overall drop-out rate did not differ substantially between depressed and non-depressed adolescents. However, participants with an adolescent subthreshold depression were more likely to drop out than the other subgroups (Alaie *et al*., 2019). Disruptive behaviour disorder was significantly associated with missingness in the registry data, while participants with low parental education and income were more likely to be missing from the follow-up assessment of depressive disorders in early adulthood (see online Supplementary Tables S1 and S2).

### Generalisability

Registry data on a reference population comprising the corresponding age group (born in 1975 and 1976) of the total Swedish population (*N* ≈ 210 000) and their parents were obtained from Statistics Sweden. Earnings were marginally higher in Uppsala County than at the national level, both for this specific age group and their parents.

### Statistical methods

For pairwise comparison of subject characteristics between groups, Fisher's exact test was used for dichotomous variables, Mantel–Haenszel chi square test was used for ordered categorical variables, and Mann–Whitney *U*-test was used for continuous variables (not normally distributed).

The association of adolescent depression with earnings in adulthood was analysed with generalised estimating equations (GEE), using a linear model to estimate the mean difference in earnings between depression groups and a log-linear quasi-Poisson model to estimate the ratio of mean earnings. A first order autoregressive correlation structure and robust standard errors were used. Adjusted analyses were performed to account for the following potential confounders: disruptive behaviour disorders, childhood and adolescent anxiety disorders, parental educational level and parental income. For descriptive purposes, we also estimated the 25th, 50th and 75th percentiles of earnings by study group and calendar year. Since sex differences in earnings are well-documented, all primary analyses were conducted separately for males and females.

The indirect (mediated) effect of depression in early adulthood (ages 19–30) on earnings in mid-adulthood (ages 31–40) was estimated in a mediation analysis. These analyses were contingent on a significant adjusted association between a specific clinical subtype of adolescent depression and earnings, and a significant association between the clinical subtype and the mediator. Since only PDD met these criteria, no mediation analyses were performed for the MDD and subthreshold groups. Analyses were performed both unadjusted and adjusted for the different confounders stated above. Standard errors for the total, direct and indirect effects were computed by non-parametric bootstrap, using 500 bootstrap replicates.

Missing data were assumed to be missing at random and were handled using multiple imputation by chained equations by specifying appropriate conditional distributions for the variables to be imputed. Thus, 25 imputed data sets were generated. Earnings were imputed using predictive mean matching with preceding and succeeding earnings, depression group and the confounders stated above as auxiliary variables. Depression in early adulthood was imputed by logistic regression, using the same set of auxiliary variables. Sensitivity analyses were performed using complete cases.

All statistical tests were two-sided and performed at the 5% significant level. The statistical analyses were performed by an external statistician (HI) with SAS version 9.4 (Cary, North Carolina, USA), using the MI and MIANALYZE procedures for multiple imputation and the GENMOD procedure for estimation using GEE.

## Results

### Description of study population

A total of 539 individuals (79% females) were included in the analyses. Child or adolescent anxiety disorders and disruptive behaviour disorders were significantly more prevalent among females and males with PDD and MDD, compared with non-depressed peers. Disruptive behaviour disorders, but not anxiety disorders, were also significantly more prevalent in females and males with subthreshold depression. Parental income was significantly lower among males with PDD and among females with MDD, compared with non-depressed peers. No significant differences between the study groups were found regarding parental education (Table 1).

**Table 1.** Characteristics of study population

	Total (*n* = 539)			PDD (*n* = 175)			MDD (*n* = 82)			Subthreshold depression (*n* = 64)			No depression (*n* = 218)		
Sex	All	Female	Male	All	Female	Male	All	Female	Male	All	Female	Male	All	Female	Male
Sex, no. (%)	539 (100)	425 (78.8)	114 (21.2)	175 (100)	140 (80.0)	35 (20.0)	82 (100)	68 (82.9)	14 (17.1)	64 (100)	46 (71.9)	18 (28.1)	218 (100)	171 (78.4)	47 (21.6)
Age at screening, mean (SD), years^a^	16.4 (0.6)	16.4 (0.6)	16.4 (0.7)	16.5 (0.6)	16.5 (0.6)	16.5 (0.7)	16.4 (0.6)	16.4 (0.6)	16.6 (0.8)	16.4 (0.7)	16.4 (0.6)	16.4 (0.8)	16.4 (0.6)	16.4 (0.6)	16.3 (0.7)
Childhood anxiety disorder, no. (%)^b^	177 (32.8)	143 (33.6)	34 (29.8)	105 (60.0)	87 (62.1)	18 (51.4)	34 (41.5)	25 (36.8)	9 (64.3)	6 (9.4)	4 (8.7)	2 (11.1)	32 (14.7)	27 (15.8)	5 (10.6)
Disruptive behaviour disorder, no. (%)^c^	112 (20.8)	78 (18.4)	34 (29.8)	60 (34.3)	43 (30.7)	17 (48.6)	20 (24.4)	13 (19.1)	7 (50.0)	16 (25.0)	10 (21.7)	6 (33.3)	16 (7.3)	12 (7.0)	4 (8.5)
Low parental education, high school or less, no. (%)^d^	271 (50.3)	220 (51.7)	51 (44.8)	82 (46.8)	69 (49.3)	13 (37.2)	50 (61.0)	42 (61.8)	8 (57.1)	35 (54.7)	25 (54.4)	10 (55.6)	104 (47.7)	84 (49.2)	20 (42.5)
Parental income in 1990, USD, mean, (SD)^e^	26 335 (11 172)	26 457 (11 717)	25 879 (8884)	25 753 (8888)	26 190 (8842)	24 002 (8984)	24 873 (9987)	24 346 (10 425)	27 434 (7268)	24 821 (10 140)	25 542 (9741)	22 978 (11 172)	27 795 (13 235)	27 760 (14 378)	27 923 (7887)

a No significant differences between subgroups.

b All comparisons between subgroups significant in both sexes except for no dep *v*. subthreshold dep. in both sexes and MDD *v*. PDD for males.

c Comparisons between subgroups significant in both sexes except for MDD *v*. subthreshold dep., MDD *v*. PDD, and subthreshold *v*. PDD in both sexes.

d No significant differences between subgroups.

e No significant differences between subgroups except for no dep *v*. MDD for females and no dep. *v*. PDD for males.

### Earnings

Earnings for the years 1996–2016 were lower for the group with a history of adolescent depression than for their non-depressed peers, with an adjusted ratio of mean earnings of 0.89 (0.82–0.97) (see online Supplementary Table S3). The most distinct difference was observed between the group with a history of PDD and the non-depressed peers, with an adjusted ratio of mean earnings of 0.85 (0.77–0.95) for females and 0.76 (0.60–0.95) for males. A similar pattern was seen for subthreshold depression, although the adjusted estimates did not reach statistically significance. The group with a history of adolescent MDD did not differ from the non-depressed group, with adjusted estimates of 0.99 (0.86–1.15) for females and 1.19 (0.92–1.53) for males (Table 2). In the adjusted model, there was no significant gender-by-subgroups interaction (*p* = 0.23).

**Table 2.** Ratio of mean earnings between analysis groups, years 1996–2016

Variable		Ratio of mean earnings (95% CI) *p*-value					
		All		Females		Males	
		Unadjusted	Adjusted^a^	Unadjusted	Adjusted^b^	Unadjusted	Adjusted^b^
Depression in adolescence	PDD *v*. no depression	0.79 (0.72–0.87) <0.001	0.83 (0.75–0.92) <0.001	0.84 (0.75–0.92) <0.001	0.85 (0.77–0.95) 0.004	0.66 (0.54–0.82) 0.001	0.76 (0.60–0.95) 0.02
	MDD *v*. no depression	0.97 (0.86–1.10) 0.68	1.02 (0.90–1.17) 0.73	0.96 (0.84–1.11) 0.61	0.99 (0.86–1.15) 0.94	1.07 (0.85–1.33) 0.58	1.19 (0.92–1.53) 0.18
	Subthreshold depression *v*. no depression	0.84 (0.74–0.95) 0.007	0.87 (0.77–0.98) 0.02	0.85 (0.75–0.97) 0.01	0.89 (0.79–1.01) 0.06	0.76 (0.58–1.01) 0.06	0.79 (0.61–1.03) 0.09
Confounding factors	Female sex	N/A	0.78 (0.71–0.86) <0.001	N/A	N/A	N/A	N/A
	Disruptive behaviour disorder	N/A	0.84 (0.76–0.92) <0.001	N/A	0.83 (0.75–0.92) <0.001	N/A	0.87 (0.71–1.06) 0.17
	Childhood anxiety disorders	N/A	1.03 (0.95–1.13) 0.48	N/A	1.06 (0.96–1.16) 0.24	N/A	0.88 (0.72–1.07) 0.21
	Parental education, college or university *v*. high school or lower	N/A	1.03 (0.95–1.11) 0.52	N/A	1.08 (0.99–1.18) 0.10	N/A	0.90 (0.76–1.06) 0.21
	Parental income, by 10 000 USD increase	N/A	1.05 (1.01–1.10) 0.02	N/A	1.05 (1.00–1.08) 0.04	N/A	1.05 (0.94–1.17) 0.43

N/A, not available.

a Adjusted for sex, disruptive behaviour disorder, childhood anxiety disorder, parental education level and parental income.

b Adjusted for disruptive behaviour disorder, childhood anxiety disorder, parental education level and parental income.

**Fig. 2.**
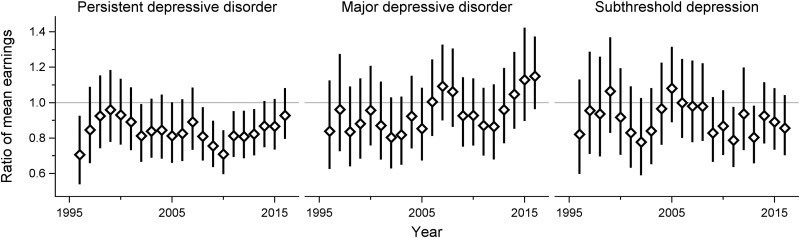
Adult earnings in females with different subtypes of adolescent depression, compared with their non-depressed counterparts. Adjusted for disruptive behavior disorders, childhood anxiety disorders, parental education level and income.

**Fig. 3.**
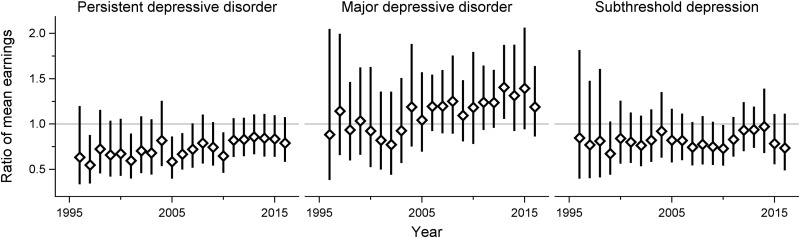
Adult earnings in males with different subtypes of adolescent depression, compared with their non-depressed counterparts. Adjusted for disruptive behavior disorders, childhood anxiety disorders, parental education level and income.

The earnings steadily increased over time throughout the study period across all subgroups. Using log-linear models, there were no significant interactions between clinical subtypes of adolescent depression and time, implying that the ratio of mean earnings between the groups did not change over time (Figs 2 and 3). In absolute terms, on the other hand, there was a gradually widening gap in earnings between both females and males with adolescent PDD or subthreshold depression and their same-sex peers without a history of depression in adolescence. However, the earnings of the MDD group did not display such a pattern (see online Supplementary Figs S1 and S2).

### Distribution

Differences in mean earnings between the PDD group and the non-depressed peers were more pronounced in the lower percentiles of the distribution. At the 25th percentile, the earnings of the females with adolescent PDD were about 25% lower compared with the non-depressed females (see online Supplementary Fig. S3). Similarly, the difference between the non-depressed males and the males with a history of PDD was particularly salient in the lower percentiles (see online Supplementary Fig. S4). A similar pattern of earnings was more indistinct in the subthreshold and MDD groups (online Supplementary Figs S3 and S4).

### Depression in early adulthood

The association of PDD with earnings in adulthood was partially mediated by depression in early adulthood (48%), after adjustment for comorbidities and parental SES (see online Supplementary Table S4). While the direct and indirect effects were similar in magnitude, only the indirect effect was statistically significant in the adjusted analyses. In gender-specific sub-analyses among those with PDD, the proportion mediated was estimated to 61% for females and 29% for males. While the estimated indirect effect was similar in both sexes, the effect reached statistical significance only in females (see online Supplementary Tables S5 and S6).

### Sensitivity analyses

All analyses were rerun using complete cases only. Overall, the same pattern of results emerged (see online Supplementary Tables S7–S11).

## Discussion

This longitudinal study sheds new light on the association between adolescent depression and subsequent earnings, defined as the sum of gross income from employment and the net income from active business. Adolescent PDD was robustly linked to reduced adult earnings in both females and males, while briefer depressive episodes were not. The difference in earnings was largely unchanged by adjustments for major child and adolescent psychiatric comorbidities and parental SES, and did not seem to mitigate over time. Indeed, the absolute difference gradually increased as the general earnings increased with age. The difference in earnings was more pronounced in the lower percentiles of the distribution. Further, the association was partially mediated by depression in early adulthood. These new insights can help inform policymaking and treatment planning.

The data utilised in this study allowed us to analyse long-term economic outcomes in novel ways. The baseline diagnostic assessment, in combination with the relatively large number of participants with a history of depressive disorder, made it possible to study the outcome across clinical subtypes of adolescent depression. Some previous studies indicate that adolescent depression reduces earnings (Fletcher, 2013; Evensen *et al*., 2017), while other studies suggest that the association might be partially or fully explained by confounding factors (Weissman *et al*., 1999; McLeod *et al*., 2016). The inconsistency of previous results indicates that there are important sources of heterogeneity. Depression is a highly heterogeneous disorder in and of itself, and our results clearly show that diagnostic differentiation is crucial to understand the long-term economic outcome. While the duration of the depressive episode emerges as a major source of heterogeneity, our results also indicate that other factors are at play. The widening gap observed in the lower percentiles of the earnings distribution suggest that some, but not all, adolescents with PDD subsequently will struggle on the labour market. The mediation analyses help clarify the pathways further, by showing that the long-term course of the depressive disorder has an impact on the outcome. In addition, the reliable year-by-year data on earnings from early to middle adulthood sets the current study apart from previous studies. The Swedish government-administered registries minimise selection bias (Ludvigsson *et al*., 2019), and sidesteps other major sources of bias (e.g. recall bias and unwillingness to report potentially sensitive information) (Evensen *et al*., 2017). Third, we were able to investigate the effect of major psychiatric comorbidities on earnings and to adjust for parental SES.

There are at least two plausible explanations for the relative difference in earnings observed here. First, early-onset depression can have a negative effect on education and other early-life milestones (Clayborne *et al*., 2019), which in the long run can lead to lower pay and increased risk for unemployment. This underscores the importance of strengthening the human capital accumulation in this group of adolescents, to avoid the penalty of reduced productivity in adulthood, as previous literature has concluded (Johar and Truong, 2014). Second, the continuity of depression and other mental health conditions into adulthood adds to the burden, which is demonstrable from our mediation analyses. Recurring depressive episodes might lead to high levels of sickness absence and make it difficult to hold a job. This notion is supported by previous research, showing that reduced productivity due to mental health conditions can have a negative effect on labour market outcomes both directly and in the long run (Chatterji *et al*., 2011; Clayborne *et al*., 2019).

To better understand the generalisability of our findings, some contextual factors must be considered. Sweden is still a country with a relatively low level of income inequality, although the income inequality has increased since the early 1990s (OECD, 2015). However, it should be noted that measures of income inequality combine earnings, self-employment and capital income, and public cash transfers. In our view, the general pattern of earnings reported in the current study is likely to be valid across time and societies although the magnitude of the relative difference in earnings might vary depending on economic and political circumstances. Factors such as skills demanded on the labour market, possibilities for life-long learning, and employment protection of permanent and temporary employments could potentially moderate the effect of early-onset depression on future earnings. There is a relatively high level of skills required on the Swedish labour market (Swedish Public Employment Service, 2019). While there might be job opportunities for those with low or incomplete education, the pay is often substantially lower. On the other hand, the demand for skills on the Swedish labour market might to some extent be counterbalanced by the fact that higher education is free of charge. Finally, it should be noted that access to mental health services could mitigate the long-term course of depressive disorders and their comorbidities. The importance of timely and effective treatment is evident from our mediation analyses. The Swedish healthcare system is to a large part publicly funded and locally managed, and has been reported to perform well on most quality indicators (OECD, 2013). Thus, the economic consequences of adolescent depression are potentially more severe in countries with more limited access to high-quality healthcare.

These contextual aspects notwithstanding, our contribution clarifies the importance of clinical characteristics such as duration and continuity for the long-term outcome of adolescent depression. Evidently, differentiation between PDD and episodic MDD in adolescence is of high relevance for economic outcomes. This observation provides further support for the recent re-classification of depressive disorders in DSM-5 (American Psychiatric Association, 2013), where PDD was introduced. Similarly, continuity of depression into adulthood seems to influence future earnings negatively. On the other hand, subthreshold depression in adolescence seemed to be linked to reduced earnings, despite the fact that this condition was not significantly associated with subsequent depressive episodes in early adulthood (Jonsson *et al*., 2011). Thus, it is possible that depressive symptoms below the threshold of MDD in some cases reflect other forms of exposure associated with reduced future earnings.

### Limitations

Some limitations should be noted. First, the controls were recruited from the same upper-secondary programmes as the depressed adolescents. Given that the prevalence of depression was higher in educational programmes with lower expected future income, this might have resulted in an underestimation of the true difference (Olsson and von Knorring, 1999). Second, despite the use of multiple imputation we cannot rule out that the estimates were biased to some extent by the attrition occurring at different stages of the study. Missing data were clearly related to some of our observed data, but we could not verify our assumption that the data were missing at random. Third, the precision of the estimates was limited for the subthreshold group and for males in general due to the small number of participants. Fourth, the assessment of depressive episodes in early adulthood covered approximately 12 years. While a life-chart was used to enhance the participants’ recollection of depressive episodes, we cannot rule out that recall bias may have resulted in an underestimation of the mediated effect. Fifth, the generalisability is restricted by the historical context of this specific cohort.

## Implications and conclusion

The duration and continued course of adolescent depression seems to be decisive for adult earnings in both males and females. These findings underscore the need for early treatment and preventive interventions, to avert negative long-term consequences and strengthen human capital.

## Supplementary material

For supplementary material accompanying this paper visit http://dx.doi.org/10.1017/S2045796020000360. click here to view supplementary material

## Data

National regulations regarding data retrieved from the Swedish registries prevent us from sharing any data openly, due to reasons related to confidentiality and protection of human privacy.
