# Supplementary material for: Adolescent depression and subsequent earnings across early to middle adulthood: a 25-year longitudinal cohort study
Source: Epidemiol Psychiatr Sci. 2020 Apr 29;29:e123. doi: 10.1017/S2045796020000360 (PMC7214705; doi:10.1017/S2045796020000360)
Supplement: Supplementary file 1 [file S2045796020000360sup001.docx]

**Online supplements**

Table S1. Characteristics of missing data for earnings

Table S2. Characteristics of missing data for depression in young adulthood

Table S3. Ratio of mean earnings between depressed and non-depressed females and males, year 1996 to 2016

Table S4. The mediating effect of recurrent depression in early adulthood (19-30 years) on CPI-adjusted earnings in mid-adulthood (age 31-40) in females and males with a history of persistent depressive disorder in adolescence

Table S5. The mediating effect of recurrent depression in early adulthood (19-30 years) on CPI-adjusted earnings in mid-adulthood (age 31-40) in females with a history of persistent depressive disorder in adolescence

Table S6. The mediating effect of recurrent depression in early adulthood (19-30 years) on CPI-adjusted earnings in mid-adulthood (age 31-40) in males with a history of persistent depressive disorder in adolescence

Table S7. Ratio of mean earnings between depressed and non-depressed females and males, year 1996 to 2016 – complete cases

Table S8. Ratio of mean earnings between analysis groups, year 1996 to 2016 – complete cases

Table S9. The mediating effect of recurrent depression in early adulthood (19-30 years) on CPI-adjusted earnings in mid-adulthood (age 31-40) in females and males with a history of persistent depressive disorder in adolescence – complete cases

Table S10. The mediating effect of recurrent depression in early adulthood (19-30 years) on CPI-adjusted earnings in mid-adulthood (age 31-40) in females with a history of persistent depressive disorder in adolescence – complete cases

Table S11. The mediating effect of recurrent depression in early adulthood (19-30 years) on CPI-adjusted earnings in mid-adulthood (age 31-40) in males with a history of persistent depressive disorder in adolescence – complete cases

Fig. S1. Mean earnings (95% CI) of females with or without a history of depressive disorders in adolescence, year 1996 to 2016

Fig. S2. Mean earnings (95% CI) of males with or without a history of depressive disorders in adolescence, year 1996 to 2016

Fig. S3. Ratio of mean earnings between females with a history of depressive disorders and non-depressed peers at 25^th^, 50^th^ and 75^th^ percentile of the distribution in each group, year 1996 to 2016

Fig. S4. Ratio of mean earnings between males with a history of depressive disorders and non-depressed peers at 25^th^, 50^th^ and 75^th^ percentile of the distribution in each group, year 1996 to 2016

**Table S1. Characteristics of missing data for earnings**

| **Total (n=539)** | **Total (n=539)** | | **Persistent depressive disorder (PDD) (n=175)** | | **Major depressive disorder (MDD) (n=82)** | | **Subthreshold depression (n=64)** | | **No depression (n=218)** | |
| --- | --- | --- | --- | --- | --- | --- | --- | --- | --- | --- |
|  | Complete data  n=472 | Incomplete data  n=67 | Complete data  n=148 | Incomplete data  n=27 | Complete data  n=68 | Incomplete data  n=14 | Complete data  n=53 | Incomplete data  n=11 | Complete data  n=203 | Incomplete data  n=15 |
| Sex, No. females (%)^a^ | 371 (78.6) | 54 (80.6) | 120 (81.1) | 20 (74.1) | 56 (82.4) | 12 (85.7) | 37 (69.8) | 9 (81.8) | 158 (77.8) | 13 (86.2) |
| Age at screening, mean (SD), y^a^ | 16.4 (0.6) | 16.4 (0.6) | 16.5 (0.6) | 16.4 (0.6) | 16.4 (0.7) | 16.4 (0.5) | 16.4 (0.7) | 16.5 (0.7) | 16.4 (0.6) | 16.5 (0.6) |
| Childhood or adolescent anxiety disorder, No. (%)^a^ | 154 (32.6) | 23 (34.3) | 91 (61.5) | 14 (51.9) | 28 (41.2) | 6 (42.9) | 4 (7.5) | 2 (18.2) | 31 (15.3) | 1 (6.7) |
| Disruptive behavior disorder, No. (%)^b^ | 88 (18.6) | 24 (35.8) | 46 (31.1) | 14 (51.9) | 18 (26.5) | 2 (14.3) | 10 (18.9) | 6 (54.5) | 14 (6.9) | 2 (13.3) |
| Low parental education, high school or less, No. (%)^a^ | 242 (51.3) | 19 (28.4) | 71 (48.0) | 11 (40.7) | 43 (29.1) | 7 (50.0) | 29 (54.7) | 6 (54.6) | 99 (48.8) | 5 (33.4) |
| Parental income (USD), mean, (SD)^a^ | 26565 (11164) | 24708 (11178) | 26242 (8874) | 23074 (8638) | 23915 (8651) | 29529 (14409) | 25179 (10106) | 23098 (10618) | 28051 (13306) | 24330 (12107) |

^a^No significant differences between incomplete and complete data. ^b^ Significant differences between incomplete and complete data for the total sample, PDD and subthreshold subgroup.

**Table S2. Characteristics of missing data for depression in young adulthood**

| **Total (n=539)** | **Total (n=539)** | | **Persistent depressive disorder (PDD) (n=175)** | | **Major depressive disorder (MDD) (n=82)** | | **Subthreshold depression (n=64)** | | **No depression (n=218)** | |
| --- | --- | --- | --- | --- | --- | --- | --- | --- | --- | --- |
|  | Complete data  n=351 | Incomplete data  n=188 | Complete data  n=115 | Incomplete data  n=60 | Complete data  n=59 | Incomplete data  n=23 | Complete data  n=30 | Incomplete data  n=34 | Complete data  n=147 | Incomplete data  n=71 |
| Sex, No. females (%)^a^ | 283 (80.6) | 142 (75.5) | 93 (80.9) | 47 (78.3) | 50 (84.7) | 18 (78.3) | 22 (73.3) | 24 (70.6) | 118 (80.3) | 53 (74.6) |
| Age at screening, mean (SD), y^a^ | 16.4 (0.6) | 16.5 (0.6) | 16.4 (0.6) | 16.6 (0.6) | 16.4 (0.6) | 16.5 (0.6) | 16.5 (0.6) | 16.3 (0.7) | 16.4 (0.6) | 16.4 (0.6) |
| Childhood anxiety disorder, No. (%)^a^ | 123 (35.0) | 54 (28.7) | 73 (63.5) | 32 (53.3) | 27 (45.8) | 7 (30.4) | 2 (6.7) | 4 (11.8) | 21 (14.3) | 11 (15.5) |
| Disruptive behavior disorder, No. (%)^a^ | 67 (19.1) | 45 (23.9) | 36 (31.3) | 24 (40.0) | 15 (25.4) | 5 (21.7) | 7 (23.3) | 9 (26.5) | 9 (6.1) | 7 (9.9) |
| Low parental education, high school or less, No. (%)^b^ | 163 (46.5) | 108 (57.4) | 46 (40.0) | 36 (60.0) | 36 (61.0) | 14 (60.8) | 14 (46.7) | 21 (61.8) | 67 (45.6) | 37 (52.1) |
| Parental income (USD), mean, (SD)^c^ | 27280 (11280) | 24570 (10778) | 26940 (7804) | 23478 (10358) | 24455 (8871) | 25946 (12575) | 24630 (9180) | 24990 (11053) | 29220 (14175) | 24846 (10527) |

^a^ No significant differences between incomplete and complete data. ^b^ Significant differences between incomplete and complete data for the total sample and PDD subgroup. ^c^ Significant differences between incomplete and complete data for the total sample, PDD subgroup and the non-depressed group.

**Table S3. Ratio of mean earnings between depressed and non-depressed females and males, year 1996 to 2016**

| **Variable** | | **Ratio of mean earnings (95% CI) p-value** | | | | | |
| --- | --- | --- | --- | --- | --- | --- | --- |
|  |  | **All** | | **Females** | | **Males** | |
|  |  | **Unadjusted** | **Adjusted^a^** | **Unadjusted** | **Adjusted^b^** | **Unadjusted** | **Adjusted^b^** |
|  | **Depression during adolescence** | 0.85 (0.78 - 0.92) <.001 | 0.89 (0.82 - 0.97) .009 | 0.87 (0.80 - 0.96) .003 | 0.90 (0.82 - 0.99) .04 | 0.77 (0.65 - 0.92) .003 | 0.84 (0.70 - 1.02) .08 |
| **Confounding factors** | **Female sex** | N/A | 0.78 (0.71 - 0.86) <.001 | N/A | N/A | N/A | N/A |
|  | **Disruptive behavior disorder** | N/A | 0.83 (0.75 - 0.91)  <.001 | N/A | 0.82 (0.73 - 0.91)  <.001 | N/A | 0.88 (0.71 - 1.08) .22 |
|  | **Childhood anxiety disorder** | N/A | 1.02 (0.94 - 1.11) .63 | N/A | 1.04 (0.95 - 1.14) .37 | N/A | 0.94 (0.78 - 1.13) .50 |
|  | **Parental education,**  **College or university vs High school or lower** | N/A | 1.02 (0.94 - 1.10) .70 | N/A | 1.07 (0.98 - 1.17) .12 | N/A | 0.86 (0.72 - 1.02) .08 |
|  | **Parental income,**  **By 10,000 USD increase** | N/A | 1.05 (1.01 - 1.09) .020 | N/A | 1.04 (1.00 - 1.08) .04 | N/A | 1.08 (0.96 - 1.21) .21 |

Note: N/A = not available

^a^ Adjusted for sex, disruptive behavior disorders, childhood anxiety disorders, parental education level, and income.

^b^ Adjusted for disruptive behavior disorders, childhood anxiety disorders, parental education level, and income.

**Table S4. The mediating effect of recurrent depression in early adulthood (19-30 years) on CPI-adjusted earnings in mid-adulthood (age 31-40) in females and males with a history of persistent depressive disorder in adolescence**

| **Depression status during adolescence** | **Depression during early adulthood** | | **Ratio of mean earnings (95% CI) / p-value**  **compared to non-depressed peers** | | | **Proportion mediated** |
| --- | --- | --- | --- | --- | --- | --- |
|  | **Proportion (95% CI)^a^** | **Relative risk (95% CI) compared to non-depressed peers** | **Total effect** | **Direct effect** | **Indirect effect** |  |
| **Unadjusted** | | | | | | |
| **No depression (n=218)** | 0.23 (0.17; 0.32) | N/A | N/A | N/A | N/A | N/A |
| **Persistent depressive disorder (n=175)** | 0.62 (0.51; 0.76) | 2.71 (1.86; 3.93) <.001 | 0.79 (0.71 - 0.88) <.001 | 0.87 (0.77 - 0.97) .02 | 0.91 (0.86 - 0.96) .002 | 0.42 |
| **Adjusted^b^** | | | | | | |
| **No depression (n=218)** | 0.26 (0.17; 0.39) | N/A | N/A | N/A | N/A | N/A |
| **Persistent depressive disorder (n=175)** | 0.60 (0.46; 0.78) | 2.31 (1.49; 3.60) <.001 | 0.83 (0.74 - 0.93) .002 | 0.90 (0.80 - 1.03) .12 | 0.92 (0.86 - 0.98) .006 | 0.48 |

Note: N/A = not available

^a^ In adjusted analysis, all other variables are fixed at their mean levels

^b^ Adjusted for sex, disruptive behavior disorders, childhood anxiety disorders, parental education, and parental income

**Table S5. The mediating effect of recurrent depression in early adulthood (19-30 years) on CPI-adjusted earnings in mid-adulthood (age 31-40) in females with a history of persistent depressive disorder in adolescence**

| **Depression status during adolescence** | **Depression during early adulthood** | | **Ratio of mean earnings (95% CI) / p-value**  **compared to non-depressed peers** | | | **Proportion mediated** |
| --- | --- | --- | --- | --- | --- | --- |
|  | **Proportion**  **(95% CI)^a^** | **Relative risk (95% CI) compared to non-depressed peers** | **Total effect** | **Direct effect** | **Indirect effect** |  |
| **Unadjusted** | | | | | | |
| **No depression (n=171)** | 0.24 (0.17 - 0.35) | N/A | N/A | N/A | N/A | N/A |
| **Persistent depressive disorder (n=140)** | 0.63 (0.51 - 0.79) | 2.58 (1.71; 3.91) <.001 | 0.83 (0.74 - 0.94) .003 | 0.91 (0.80 - 1.03) .13 | 0.92 (0.87 - 0.98) .008 | 0.47 |
| **Adjusted^b^** | | | | | | |
| **No depression (n=171)** | 0.27 (0.17 - 0.42) | N/A | N/A | N/A | N/A | N/A |
| **Persistent depressive disorder (n=140)** | 0.63 (0.49 - 0.80) | 2.31 (1.41; 3.78) <.001 | 0.87 (0.77 - 0.98) .03 | 0.94 (0.82 - 1.08) .41 | 0.92 (0.86 - 0.98) .01 | 0.61 |
| Note: N/A = not available  ^a^ In adjusted analysis, all other variables are fixed at their mean levels.  ^b^ Adjusted for disruptive behavior disorders, childhood anxiety disorders, parental education, and parental income | | | | | | |

**Table S6. The mediating effect of recurrent depression in early adulthood (19-30 years) on CPI-adjusted earnings in mid-adulthood (age 31-40) in males with a history of persistent depressive disorder in adolescence**

| **Depression status during adolescence** | **Depression during early adulthood** | | **Ratio of mean earnings (95% CI) / p-value**  **compared to non-depressed peers** | | | **Proportion mediated** |
| --- | --- | --- | --- | --- | --- | --- |
|  | **Proportion**  **(95% CI)^a^** | **Relative risk (95% CI) compared to non-depressed peers** | **Total effect** | **Direct effect** | **Indirect effect** |  |
| **Unadjusted** | | | | | | |
| **No depression (n=47)** | 0.17 (0.07; 0.39) | N/A | N/A | N/A | N/A | N/A |
| **Persistent depressive disorder (n=35)** | 0.57 (0.35; 0.92) | 3.40 (1.33; 8.65) .010 | 0.67 (0.53 - 0.84) <.001 | 0.75 (0.59 - 0.95) .02 | 0.89 (0.78 - 1.02) .10 | 0.32 |
| **Adjusted^b^** | | | | | | |
| **No depression (n=47)** | 0.22 (0.09; 0.55) | N/A | N/A | N/A | N/A | N/A |
| **Persistent depressive disorder (n=35)** | 0.53 (0.30; 0.92) | 2.40 (0.83; 6.94) .11 | 0.71 (0.53 - 0.94) .02 | 0.77 (0.58 - 1.03) .08 | 0.92 (0.79 - 1.06) .23 | 0.29 |
| Note: N/A = not available  ^a^ In adjusted analysis, all other variables are fixed at their mean levels.  ^b^ Adjusted for disruptive behavior disorders, childhood anxiety disorders, parental education, and parental income | | | | | | |

**Table S7. Ratio of mean earnings between depressed and non-depressed females and males, year 1996 to 2016 – complete cases**

| **Variable** | | **Ratio of mean earnings (95% CI) p-value** | | | | | |
| --- | --- | --- | --- | --- | --- | --- | --- |
|  |  | **All** | | **Females** | | **Males** | |
|  |  | **Unadjusted** | **Adjusted^a^** | **Unadjusted** | **Adjusted^b^** | **Unadjusted** | **Adjusted^b^** |
|  | **Depression during adolescence** | 0.85 (0.78 - 0.92) <.001 | 0.89 (0.82 - 0.97) .01 | 0.88 (0.80 - 0.96) .004 | 0.91 (0.82 - 1.00) .04 | 0.77 (0.65 - 0.92) .004 | 0.84 (0.69 - 1.02) .07 |
| **Confounding factors** | **Female sex** | N/A | 0.78 (0.71 - 0.86) <.001 | N/A | N/A | N/A | N/A |
|  | **Disruptive behavior disorder** | N/A | 0.83 (0.75 - 0.91) <.001 | N/A | 0.82 (0.74 - 0.91) <.001 | N/A | 0.87 (0.70 - 1.08) .20 |
|  | **Childhood anxiety disorder** | N/A | 1.02 (0.94 - 1.11) .63 | N/A | 1.04 (0.95 - 1.14) .35 | N/A | 0.94 (0.78 - 1.13) .50 |
|  | **Parental education,**  **College or university vs High school or lower** | N/A | 1.02 (0.94 - 1.11) .66 | N/A | 1.08 (0.99 - 1.18) .10 | N/A | 0.86 (0.72 - 1.02) .09 |
|  | **Parental income,**  **By 10,000 USD increase** | N/A | 1.05 (1.01 - 1.09) .02 | N/A | 1.04 (1.00 - 1.09) .04 | N/A | 1.07 (0.96 - 1.21) .23 |

Note: N/A = not available

^a^ Adjusted for sex, disruptive behavior disorder, childhood anxiety, parental education level and income.

^b^ Adjusted for disruptive behavior disorder, childhood anxiety, parental education level and income.

**Table S8. Ratio of mean earnings between analysis groups, year 1996 to 2016 – complete cases**

| **Variable** | | **Ratio of mean earnings (95% CI) p-value** | | | | | |
| --- | --- | --- | --- | --- | --- | --- | --- |
|  |  | **All** | | **Females** | | **Males** | |
|  |  | **Unadjusted** | **Adjusted^a^** | **Unadjusted** | **Adjusted^b^** | **Unadjusted** | **Adjusted^b^** |
| **Depression in Adolescence** | **Persistent depressive disorder vs No depression** | 0.79 (0.72 - 0.87) <.001 | 0.83 (0.75 - 0.91)  <.001 | 0.84 (0.76 - 0.93)  <.001 | 0.85 (0.77 - 0.95) .004 | 0.65 (0.52 - 0.82)  <.001 | 0.74 (0.58 - 0.95) .02 |
|  | **Major depressive disorder vs No depression** | 0.98 (0.86 - 1.11) .71 | 1.03 (0.90 - 1.17) .69 | 0.96 (0.83 - 1.11) .61 | 1.00 (0.86 - 1.16) .98 | 1.07 (0.86 - 1.35) .53 | 1.21 (0.93 - 1.56) .15 |
|  | **Subthreshold depression vs No depression** | 0.85 (0.75 - 0.96) .009 | 0.87 (0.78 - 0.98) .02 | 0.86 (0.77 - 0.98) =0.02 | 0.90 (0.80 - 1.01) .08 | 0.76 (0.57 - 1.00) .05 | 0.79 (0.61 - 1.03) .08 |
| **Confounding factors** | **Female sex** | N/A | 0.78 (0.71 - 0.86)  <.001 | N/A | N/A | N/A | N/A |
|  | **Disruptive behavior disorder** | N/A | 0.83 (0.76 - 0.92)  <.001 | N/A | 0.83 (0.75 - 0.92)  <.001 | N/A | 0.86 (0.70 - 1.06) .17 |
|  | **Childhood anxiety disorder** | N/A | 1.04 (0.95 - 1.13) .43 | N/A | 1.06 (0.97 - 1.17) .19 | N/A | 0.87 (0.71 - 1.07) .19 |
|  | **Parental education,**  **College or university vs High school or lower** | N/A | 1.03 (0.95 - 1.12) .49 | N/A | 1.08 (0.99 - 1.19) .09 | N/A | 0.90 (0.76 - 1.07) .24 |
|  | **Parental income,**  **By 10,000 USD increase** | N/A | 1.05 (1.01 - 1.09) .021 | N/A | 1.04 (1.00 - 1.09) .04 | N/A | 1.05 (0.93 - 1.17) .44 |

Note: N/A = not available

^a^ Adjusted for sex, disruptive behavior disorder, childhood anxiety, parental education level and income.

^b^ Adjusted for disruptive behavior disorder, childhood anxiety, parental education level and income.

**Table S9. The mediating effect of recurrent depression in early adulthood (19-30 years) on CPI-adjusted earnings in mid-adulthood (age 31-40) in females and males with a history of persistent depressive disorder in adolescence – complete cases**

| **Depression status during adolescence** | **Depression during early adulthood** | | **Ratio of mean earnings (95% CI) / p-value**  **compared to non-depressed peers** | | | **Proportion mediated** |
| --- | --- | --- | --- | --- | --- | --- |
|  | **Proportion (95% CI)^a^** | **Relative risk (95% CI) compared to non-depressed peers** | **Total effect** | **Direct effect** | **Indirect effect** |  |
| **Unadjusted** | | | | | | |
| **No depression (n=147)** | 0.21 (0.15; 0.30) | N/A | N/A | N/A | N/A | N/A |
| **Persistent depressive disorder (n=115)** | 0.62 (0.49; 0.78) | 2.93 (1.92; 4.46) <.001 | 0.79 (0.70 - 0.88) <.001 | 0.86 (0.76 - 0.97) .02 | 0.92 (0.87 - 0.97) .004 | 0.38 |
| **Adjusted^b^** | | | | | | |
| **No depression (n=147)** | 0.23 (0.15; 0.36) | N/A | N/A | N/A | N/A | N/A |
| **Persistent depressive disorder (n=115)** | 0.61 (0.45; 0.82) | 2.60 (1.58; 4.28) <.001 | 0.82 (0.72 - 0.93) .002 | 0.89 (0.77 - 1.02) .10 | 0.92 (0.86 - 0.98) .008 | 0.44 |

Note: N/A = not available

^a^ In adjusted analysis, all other variables are fixed at their mean levels.

^b^ Adjusted for disruptive behavior disorders, childhood anxiety disorders, parental education, and parental income

**Table S10. The mediating effect of recurrent depression in early adulthood (19-30 years) on CPI-adjusted earnings in mid-adulthood (age 31-40) in females with a history of persistent depressive disorder in adolescence – complete cases**

| **Depression status during adolescence** | **Depression during early adulthood** | | **Ratio of mean earnings (95% CI) / p-value**  **compared to non-depressed peers** | | | **Proportion mediated** |
| --- | --- | --- | --- | --- | --- | --- |
|  | **Proportion (95% CI)^a^** | **Relative risk (95% CI) compared to non-depressed peers** | **Total effect** | **Direct effect** | **Indirect effect** |  |
| **Unadjusted** | | | | | | |
| **No depression (n=118)** | 0.23 (0.16; 0.33) | N/A | N/A | N/A | N/A | N/A |
| **Persistent depressive disorder (n=93)** | 0.62 (0.48; 0.81) | 2.73 (4.30; 1.73) <.001 | 0.83 (0.72 - 0.95) .006 | 0.89 (0.77 - 1.03) .13 | 0.93 (0.87 - 0.99) .02 | 0.41 |
| **Adjusted^b^** | | | | | | |
| **No depression (n=118)** | 0.24 (0.15; 0.38) | N/A | N/A | N/A | N/A | N/A |
| **Persistent depressive disorder (n=93)** | 0.62 (0.46; 0.83) | 2.60 (4.46; 1.52) <.001 | 0.85 (0.73 - 0.99) .04 | 0.93 (0.78 - 1.10) .37 | 0.92 (0.86 - 0.99) .02 | 0.54 |

Note: N/A = not available

^a^ In adjusted analysis, all other variables are fixed at their mean levels.

^b^ Adjusted for disruptive behavior disorders, childhood anxiety disorders, parental education, and parental income

**Table S11. The mediating effect of recurrent depression in early adulthood (19-30 years) on CPI-adjusted earnings in mid-adulthood (age 31-40) in males with a history of persistent depressive disorder in adolescence – complete cases**

| **Depression status during adolescence** | **Depression during early adulthood** | | **Ratio of mean earnings (95% CI) / p-value**  **compared to non-depressed peers** | | | **Proportion mediated** |
| --- | --- | --- | --- | --- | --- | --- |
|  | **Proportion (95% CI)^a^** | **Relative risk (95% CI) compared to non-depressed peers** | **Total effect** | **Direct effect** | **Indirect effect** |  |
| **Unadjusted** | | | | | | |
| **No depression (n=29)** | 0.14 (0.05; 0.37) | N/A | N/A | N/A | N/A | N/A |
| **Persistent depressive disorder (n=22)** | 0.59 (0.34; 1.02) | 4.28 (13.14; 1.40) .01 | 0.65 (0.47 - 0.89) .007 | 0.71 (0.55 - 0.92) .008 | 0.91 (0.72 - 1.16) .44 | 0.25 |
| **Adjusted^b^** | | | | | | |
| **No depression (n=29)** | 0.19 (0.06; 0.55) | N/A | N/A | N/A | N/A | N/A |
| **Persistent depressive disorder (n=22)** | 0.53 (0.29; 0.97) | 2.83 (10.54; 0.76) .12 | 0.71 (0.50 - 1.01) .06 | 0.74 (0.54 - 1.03) .07 | 0.95 (0.75 - 1.21) .70 | 0.16 |

Note: N/A = not available

^a^ In adjusted analysis, all other variables are fixed at their mean levels.

^b^ Adjusted for disruptive behavior disorders, childhood anxiety disorders, parental education, and parental income


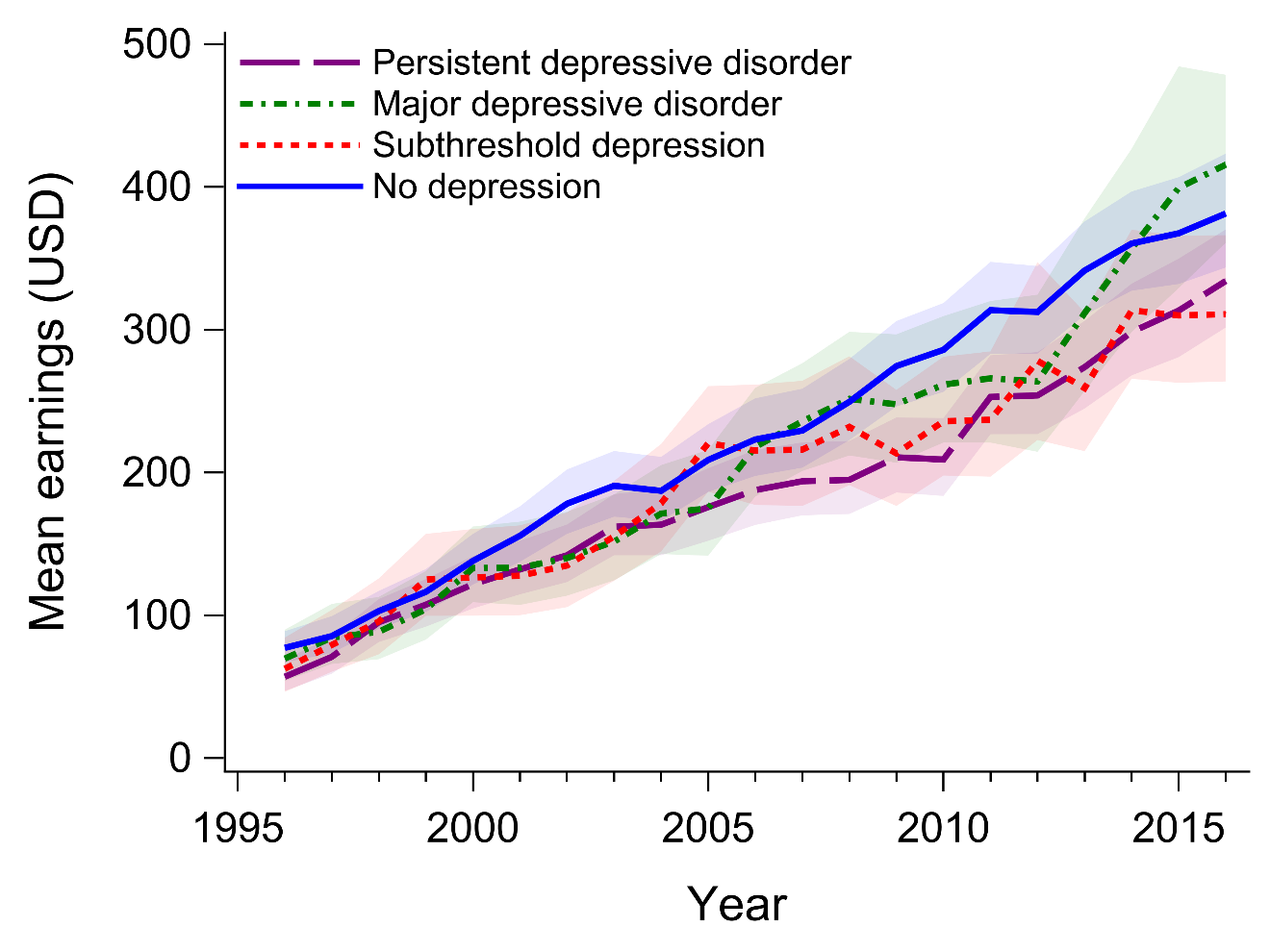


**Fig. S1. Mean earnings (95% CI) of females with or without a history of depressive disorders in adolescence, year 1996 to 2016**


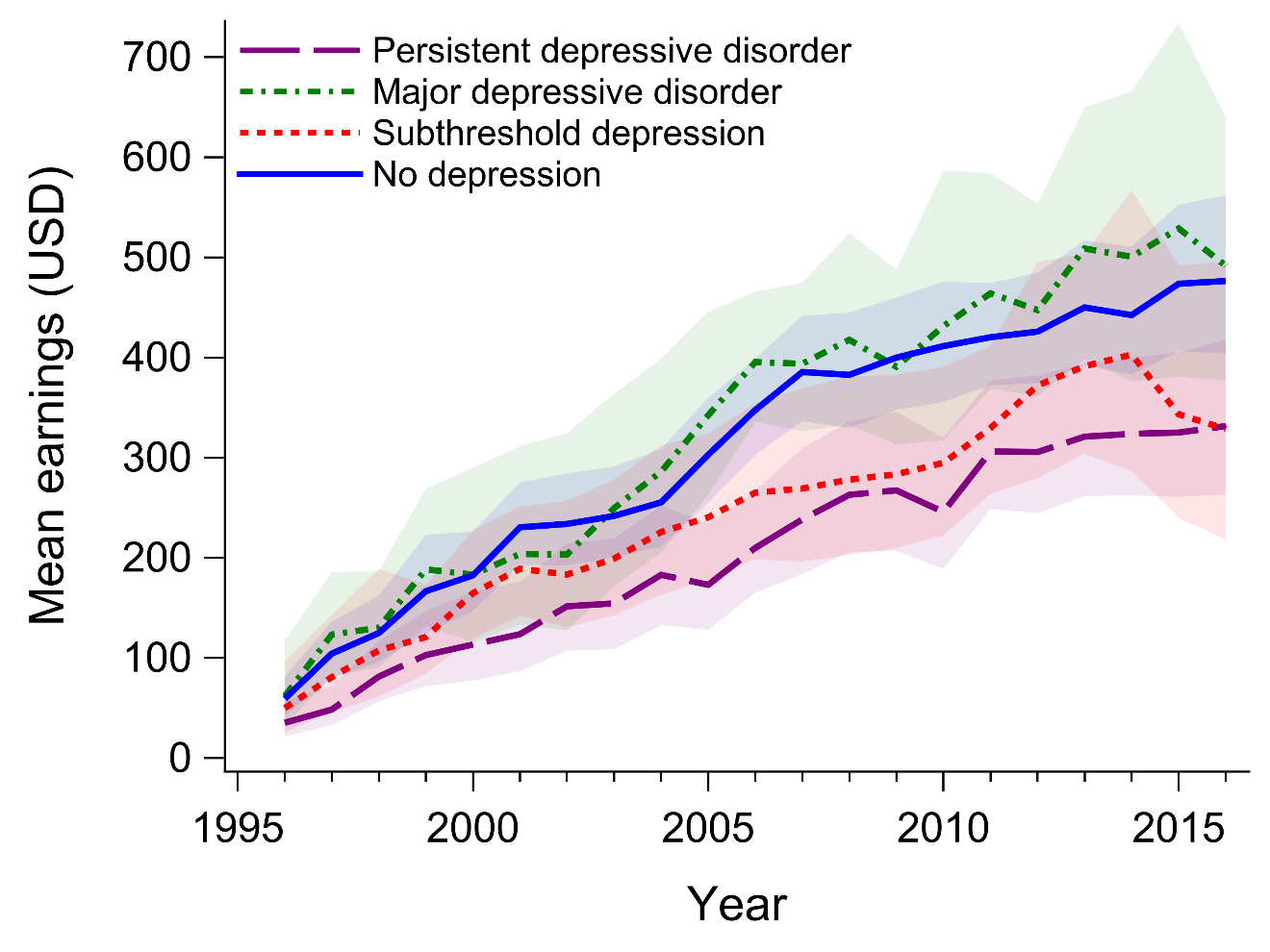


**Fig. S2. Mean earnings (95% CI) of males with or without a history of depressive disorders in adolescence, year 1996 to 2016**

|  |
| --- |

**
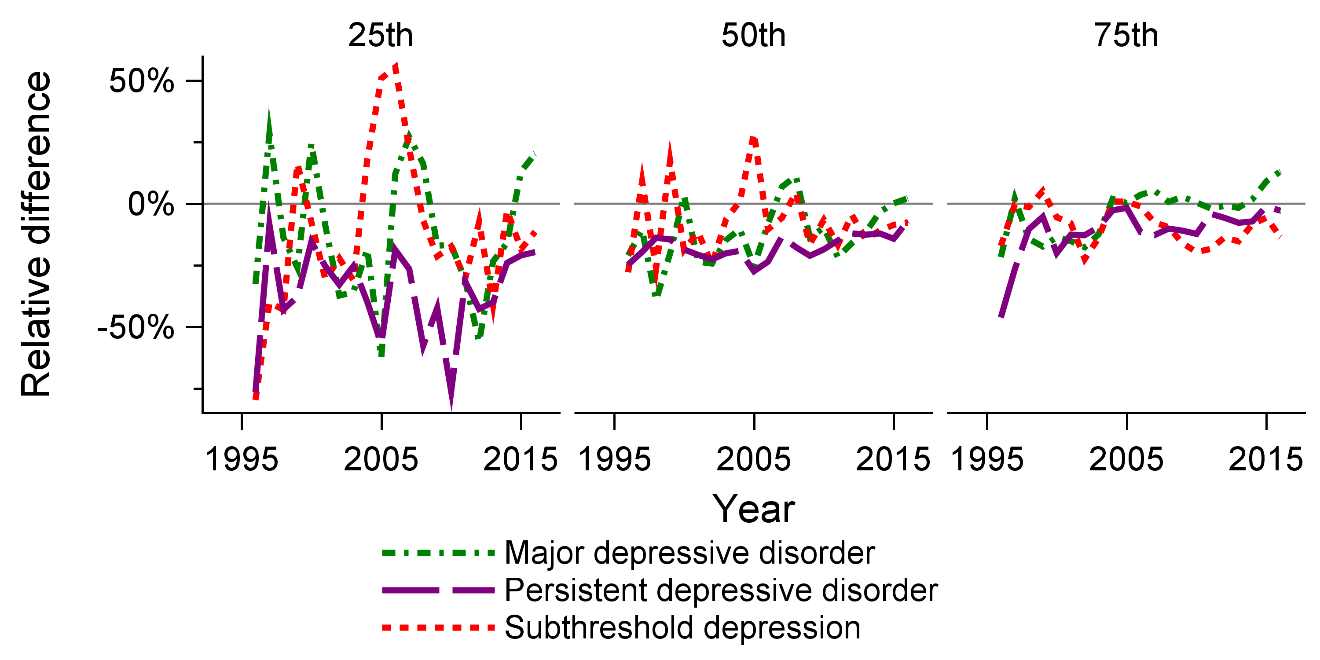
**

**Fig. S3. Ratio of mean earnings between females with a history of depressive disorders and non-depressed peers at 25th, 50th and 75th percentile of the distribution in each group, year 1996 to 2016**

|  |
| --- |

**
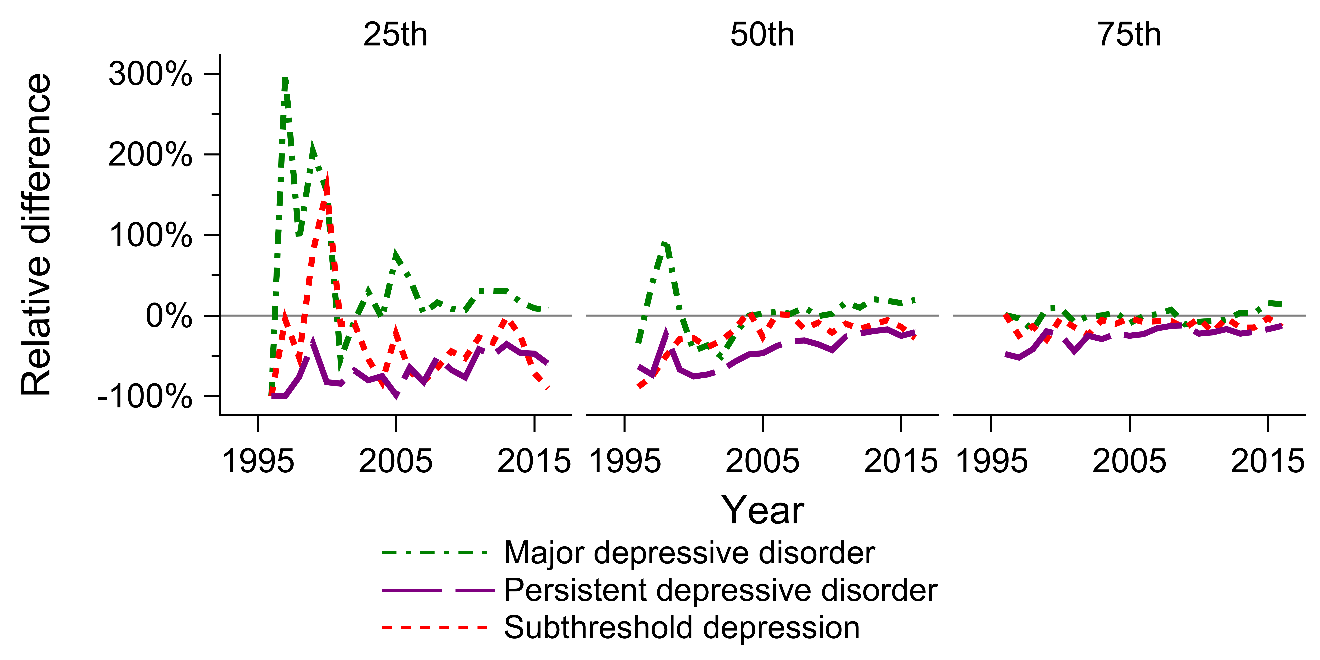
**

**Fig. S4. Ratio of mean earnings between males with a history of depressive disorders and non-depressed peers at 25th, 50th and 75th percentile of the distribution in each group, year 1996 to 2016**
